# Progress in Cancer

**Published:** 1902-07-05

**Authors:** 


### PROGRESS IN CANCER.

The numerous publications on the subject of the parasite of cancer, or cellular degeneration in that disease, have drawn from the pen of Sanfelice[1] a paper in which he endeavours to show that the writers—Nichols, Borrel, and Gaylord—have not seen the blastomycetes which he holds to be concerned in the ætiology of malignancy. Nichols, after examining 16 cases only, finds the "parasite" in 13 of them. He holds that the appearance of the blastomycetes supplied to him by Plimmer and Sanfelice, after being passed through guinea-pigs and rabbits, is not the same with this "parasite." He also maintains that cancer cannot be produced by the inoculation of the blastomycetes, Sanfelice's successes in dogs being coincidences. To this Sanfelice replies that he has had further successes in this direction, and Plimmer and Leopold, apart from him, have had similar results. Borrel's suggestion—that the "parasites" are developments of an element in the cancer cell, viz., the attraction sphere—is valueless, as the plates show forms bearing no resemblance to the true endo-cellular parasites of cancer. There are also true cellular inclusions quite different from true parasites: these are wrongly included in Borrel's degeneration forms. Borrel denies that cultures of blastomycetes can be made from cancers. Now Sanfelice shows that this can only be accomplished in those rare cases of rapidly-growing cancers which show abundant parasites. In old-standing cases the parasites show themselves as Russell's or fuchsine bodies, and are no longer cultivable, being merely chromatic residua of the blastomycetes. Dead blastomycetes, whether pathogenic or not, injected into dogs or cats reveal the same appearances. And this will explain those cases where Russell's bodies have been found in non-cancerous tumours. Gaylord's supposition, that the forms seen by him are protozoa, and not blastomycetes, arises partly from his mistaking cellular degenerations in malignant tumours for parasitic forms, and partly from a comparison between the so-called protozoa, which have not yet definitely been shown to belong to the protozoa. Sanfelice points out how certain mesodermic elements may enter cells and produce the appearance of protozoa within them. They differ greatly from the "parasites" in their ultimate condition and their staining reactions. "They appear," he says, "as hyaline areas of various shapes, with or without contents, which are stained very faintly, and which need not be confounded with the chromatic residua, etc., of a nucleus." It is for this reason that Borrel's arcoplasm theory does not explain the phenomena. Gaylord has figured many of these as parasites. In these endo-cellular forms there is no trace of a limiting membrane, and so they are different from the true parasites. They also contain at times a minute deeply-staining granule, but it is only when the protoplasmic mass contained in the interior of one of these areas is diffusely and deeply stained of a violet colour by Ehrlich's fluid and carbolfuchsine 1 to 3 of water, that these can be confused with a young blastomyces surrounded by a clear zone in a cancer cell. The blastomyces has a body and capsule—the body homogeneous, and either deeply or faintly stained; or there may be a deeper-stained portion in the middle, surrounded by a less deeply stained halo, which may be in the form of a concentric ring. When examined fresh they reveal a highly refractile membrane of variable thickness, with perhaps a hyaline membrane outside this.

While this discussion proceeds about the parasite of cancer, Doyen[2] reports the finding of a characteristic micrococcus in cancerous tumours. He has isolated the organism in pure culture, singly, in pairs, or in short chains. He terms it micrococcus neoformans. Injections into animals produce intense epithelial inflammation, and adenomata are formed. Sterilised cultures induce a local reaction in cancer cases comparable with that of tuberculin. Doyen is satisfied with results obtained after operation by injecting this material. Powell White looks to some intrinsic factor in the causation of cancer. He thinks a great deal more information might be obtained from the clinical study of the temperature, urine and the phenomena of cachexia of cancer patients. He thinks that much might be done by hygienic treatment, as in tuberculosis, and suggests the use of arsenic as a drug. In checking the growth of cancer some have looked to cutting off the blood supply. Powell White suggests that artificial glycosuria, if induced in patients suffering from malignant disease, might have the effect of removing the glycogen upon which the cancer cells feed. The success would largely depend, however, upon whether the tumour cells would part with the glycogen sooner than the normal tissues.

Beatson[3] expresses his views concisely in this way: That cancer can only develop in tissues that have special proliferating power, either natural, as in Cohnheim's "rests," or acquired in one of many possible ways. Further, the actual exciting cause of cell proliferation is a secretion from all the tissues of the body, and possessed of fructifying powers. A mutual relationship also exists between the character and amount of this secretion and the testes and ovaries. Another factor is the inherent vitality of the tissues themselves. And it is in this way alone that the disappearance of cancerous tissue under oöphorectomy can be satisfactorily explained.

That oöphorectomy alone has some good effect upon carcinoma is instanced by McAdam Eccles,[4] who has treated an inoperable carcinoma mammæ by oöphorectomy, with the result that the recurrent nodules softened and disappeared, glands retrogressed, and 16 months later the patient described herself as "perfectly well." The softening began before thyroid extract was administered.

[1] Scottish Med. and Sci. Jour., Jan.; Med. Rec., Feb. 8.
[2] Brit. Med. Jour., March 15. [3] Lancet, March 15. [4] Clin. Jour., March 19.

